# Underwater Soft Robotics: A Review of Bioinspiration in Design, Actuation, Modeling, and Control

**DOI:** 10.3390/mi13010110

**Published:** 2022-01-10

**Authors:** Samuel M. Youssef, MennaAllah Soliman, Mahmood A. Saleh, Mostafa A. Mousa, Mahmoud Elsamanty, Ahmed G. Radwan

**Affiliations:** 1 Smart Engineering Systems Research Center (SESC), Nile University, Sheikh Zayed City 12588, Egypt; melsamanty@nu.edu.eg; 2 School of Engineering and Applied Sciences, Nile University, Sheikh Zayed City 12588, Egypt; msoliman@nu.edu.eg (M.S.); mabdullah@nu.edu.eg (M.A.S.); agradwan@nu.edu.eg (A.G.R.); 3 Nile University’s Innovation Hub, Nile University, Sheikh Zayed City 12588, Egypt; mabdelrahman@nu.edu.eg; 4 Mechanical Department, Faculty of Engineering at Shoubra, Benha University, Cairo 11672, Egypt; 5 Department of Engineering Mathematics and Physics, Cairo University, Giza 12613, Egypt

**Keywords:** soft robotics, underwater robots, design, actuation, modeling, control, learning

## Abstract

Nature and biological creatures are some of the main sources of inspiration for humans. Engineers have aspired to emulate these natural systems. As rigid systems become increasingly limited in their capabilities to perform complex tasks and adapt to their environment like living creatures, the need for soft systems has become more prominent due to the similar complex, compliant, and flexible characteristics they share with intelligent natural systems. This review provides an overview of the recent developments in the soft robotics field, with a focus on the underwater application frontier.

## 1. Introduction

Underwater exploration, much like space exploration, has been at the frontier of science and engineering ventures. As with the many Mars missions, where rovers and mobile robots are deployed instead of humans, deep underwater missions are mostly carried out using underwater robots. However, to this day, delving deep within the oceans of our planet still poses many challenges for these robotic systems. Some of the early robotic systems sent by humans to explore marine life are known as remotely operated vehicles (ROVs) [1]. ROVs are underwater robots, manually operated by a pilot, using tethered communication. They mainly have a rigid body hull and are actuated using electric thrusters. Autonomous underwater vehicles (AUVs) are similar to ROVs but differ in that they are untethered and do not require a pilot or an operator, as they are programmed to autonomously perform specific tasks. Both ROVs and AUVs vary in size, depending on the type of tasks they are manufactured to perform.

These underwater robotic systems are used to execute a wide range of underwater applications such as maintenance and monitoring applications. Such applications include underwater pipe inspection, offshore infrastructure repairs, and condition monitoring. Biological applications include seabed and abyssal exploration, sample gathering from marine environments such as coral reefs, and ecological aquatic phenomena monitoring and data collection [2]. More specifically, repairing and sampling tasks are carried out using underwater vehicle manipulator systems (UVMSs). UVMSs are unmanned underwater vehicles (UUVs) such as ROVs and AUVs that are equipped with different types of underwater manipulators that are suitable for the mentioned tasks [3]. The majority of manipulators used for underwater applications are actuated using hydraulic or electric systems. They can be used for the installation and maintenance 0f infrastructure such as pipes and cables [4], salvaging debris and sunken objects, mineral exploration [5], and biological samples gathering [6].

Even with the technological advances in UUVs and UVMS, they still face several limitations that prevent deep sea exploration. For instance, AUVs are limited to long-distance maneuvering at middle depths, while the use of ROVs is challenging in surface waters with strong waves and disturbances caused by currents. ROVs and UVMS also require constant communication with the operator, which becomes harder during seabed tasks due to the limitations of tethered communication. Some proposed solutions involve remote satellite communication [7]. In addition, the use of rigid manipulators is undesired for gathering delicate marine samples such as coral reefs, as they are hard to handle and can be damaged by the manipulators.

All these rigid robots have limited flexibility and adaptability to their environment. By contrast, soft robots offer more adaptability due to their compliant nature [8]. Such adaptability is exhibited in their ability to deform and change their shape according to the surrounding environment with which they are interacting. For example, soft robots made from compliant materials can achieve shrinking and bending motion that allow them to navigate within narrow areas. They also offer the possibility to grasp and manipulate various objects by adapting to their different shapes and structures. The evolution of soft robots led to advances in the pursuit of biomimicry of living creatures. The ability of soft robots to deform, change their shapes, exhibit infinite degrees of freedom, and perform complex motion, makes them a suitable candidate for the basis of biological emulation, especially that of underwater creatures, which are one of the sources of biomimetic inspiration for robotic and engineering systems. The exploitation of theses advantages offered by soft robots would help solve some of the challenges facing deep sea exploration. For instance, the use of soft manipulators allows safe handling of delicate coral reef samples [9,10], whereas imitating fishes’ and marine creatures’ propulsion methods can offer more efficient solutions for exploration and maneuvering at depth or in perturbed surface waters [11,12,13,14].

Alongside the great potential of soft robots, many challenges need to be overcome in order to reach the full functionality of bioinspired soft intelligent systems. Key challenges vary from design and fabrication to modeling and control. These challenges and the current state of research in solving them are discussed in this review.

## 2. Underwater Locomotion

**Figure 1:**
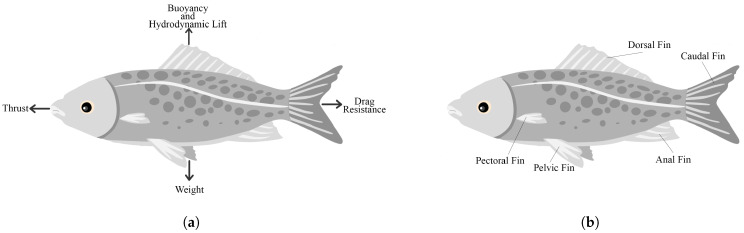
(**a**) The underwater forces acting on the fish during swimming. (**b**) Fish anatomy showing the different fins fish use to swim and stabilize.

Marine environments can seem extraterrestrial for humans at times. Hence, the study of the locomotion techniques and the morphology of aquatic creatures is essential. These types of biological studies offer insights providing keys toward the successful mimicry of these marine creatures. The aquatic environment plays a large role in defining the types of underwater locomotion, as governed by the four main forces acting on bodies underwater [15]: vertical weight and buoyancy alongside hydrodynamic lift, and horizontal thrust and drag (Figure 1a). Fish are able to generate lift and thrust in order to swim. They can achieve swimming using their fins or swimming propulsors (Figure 1b). According to the motion of these fins, fish swimming methods can be classified into several categories.

The two main categorizations of fish motion are based on which fins are performing the bending motion and the frequency at which the fins move. In terms of the first category, fish use their body and/or caudal fin to generate thrust (BCF). Examples include carangiform and anguilliform such as tuna and eel. Other types of fish use their median and/or paired fins (MPF). Examples include rajiform and labriform such as batoids. The frequency of movement of the fish’s body and fins indicates whether the motion is undulatory or oscillatory. During undulatory motion, the fish’s body performs a wave-shaped pattern, whereas oscillatory swimming uses only swivel-like motion.

Additional underwater locomotion modes fall outside the previous categorizations [16]. One example is the jet propulsion performed by jellyfish, octopus, and squid. Drag-induced swimming is exhibited by turtles as they generate thrust by moving their flippers in the opposing direction of motion. Friction-based crawling is performed by crustaceans, and echinoderms such as starfish use adhesive-based crawling.

In terms of assessing swimming performance, one of the most important metrics is the swimming speed of fish and, in particular, the critical swimming speed ($U_{c}rit$), which is commonly measured in centimeters per second (cm/s) or body lengths per second (BL/s) [17,18]. One of the main factors that affects fish swimming speed is the tail beat frequency in Hertz (Hz). It relates to the fish’s velocity through the stride length, which is the distance traveled by the fish per tail beat, expressed as ratio of the body length (L) [19,20]. The Reynolds number (Re) and Strouhal number (St) are also important factors to assess the hydrodynamic performance of the fish’s swimming. Several robotic fish platforms inspired from actual fish morphology and swimming, such as tuna, use the same metrics to assess their robots’ performance [13,21,22,23]. Another important factor is to analyze the efficiency of fish propulsion. However, it is hard to establish an accurate measure of propulsive efficiency for real biological fish. In general, efficiency is defined as the ratio of useful output to total input. For a self-propelled body, the measure of such work depends on the drag the body needs to overcome to move, which is hard to quantify as it differs with the shape of the body, as well as the body-propulsor hydrodynamics [24]. It is also challenging to determine input power in fish, which relates to muscle shaft power and the fish’s metabolism and oxygen (fuel) consumption [25]. A common metric used to quantify the fitness of fish and their efficiency is the cost of transport (COT), defined as the energy expended per traveled distance. The COT is a good indication of the fish’s swimming efficiency, and there have been several attempts to define and normalize COT for fish propulsive efficiency [24,26,27,28].

## 3. Challenges and Potentials of Soft Robots

### 3.1. Design

#### 3.1.1. Bioinspiration

Since its inception, the field of robotics has drawn inspiration from nature. The main aspect of nature imitation in robotics is apparent in the design and structure of the robots’ bodies that aim to mimic biological systems. By looking at the knowledge gained through biomechanics studies, living creatures with mobile abilities are mainly classified into two groups based on their body structure: vertebrates and invertebrates. Vertebrates include fish, mammals, birds, amphibians, and reptiles; invertebrates include crustaceans (crab, lobster), echinoderms (starfish, sea urchin), coelenterates (jellyfish), arachnids, molluscs (octopus, squid), insects, and worms, among others [29].

The challenge of building robotic systems with motion capabilities similar to those of these creatures lies in their body construction, which exhibits compliance ranging from only a few parts such as an elephant’s trunk or mammals’ organs, to completely soft and deformable bodies in the case of some invertebrates such as jellyfish. The main contributor to this compliance is the elastic nature of the building blocks of these bodies such as muscles, tendons, skin, tissues, and cartilages, as they are known for having low Young’s modulus (less than one gigapascal) [8].

Some attempts have been made to mimic some of these animals using hard materials. However, due to the limited degrees of freedom offered by conventional rigid robots compared to the infinite degrees and redundancy of soft bodies, different structures with continuum deformations had to be implemented. In contrast to conventional non-redundant rigid robots, discrete hyper-redundant and hard continuum robots offer large to infinite degrees of freedom, which brings them closer to mimicking vertebrates’ motion [30,31]. Common examples include tendon-driven continuum manipulators [32,33,34]. One of the first underwater robots to employ a structure of discrete multiple rigid-link sections actuated by tendons is the RoboTuna robotic fish [35]; The VCUUV prototype, inspired by RoboTuna, uses hydraulic actuation to drive an articulated tail [36]. Other serial multi-joint biomimetic fish robots have been developed to imitate carangiform swimming [37,38].

Despite providing more degrees of freedom than rigid robots, hard continuum robots still lack the shape adaptability offered by soft robots, which would help bring robots closer to their bioinspired creatures. The Compliant Robotic Tuna (CRT) [39] is an example of a biomimetic fish robot having a servo-actuated compliant body and tail and is able to perform swimming maneuvers. The Soft Robotic Fish (SoFi) [40] is a marine exploration robot capable of 3D swimming that imitates fish motion. It is driven by a soft fluidic actuator and has a buoyancy control unit for depth adjustment. Other marine creatures such as batoids were also mimicked, as in the case of the stingray robot with a soft silicone outer body and pectoral fins [41].

#### 3.1.2. Design Optimization

Even when taking inspiration from nature, designing soft robots with the desired mechanical behaviors that allow them to perform specific tasks presents another challenge. The complexity of such robotic systems, due to their unconventional components from materials to actuation, makes it hard to use currently known design and simulation tools to build soft robots [42,43]. Optimization techniques have been proposed to help automate the design process, and bridge the gap between simulation, fabrication, and the actual performance of soft robots. The general optimization framework can be summarized as choosing the design behavior to be optimized, such as crawling or grasping; identifying the design variables to be optimized, such as the material and the actuation; and defining the constraints of the system. The optimization process iteratively evaluates the design candidates using analysis tools and searches for the optimal design.

One approach uses evolutionary optimization algorithms to automate the design and manufacturing of freeform soft robots. This approach uses voxel-based dynamic simulation to evaluate the morphology and locomotion of the robot [44]. Voxels are soft cubic blocks with specific parameters, such as stiffness and Poisson’s ratio, that undergo volumetric change when forces are applied to them. Another voxel-based method aims to optimize the morphology to achieve adaptability using the property of criticality, which allows the robot to perform more diverse tasks [45].

Another conceptual design approach provides a spatial grammar to build soft robots and optimize their design for locomotion and actuation [46]. The spatial grammar generates sub-assemblies of interconnected balls based on a set of defined rules. The generated models are then evaluated and optimized in terms of locomotion abilities.

Performing design optimization for underwater soft robots is an even more challenging problem, as the effect of the environment on the robot’s morphology needs to be taken into account. DiffAqua [47], a computational design pipeline, relies on differentiable simulation to perform gradient-based optimization for the geometry and control of soft underwater swimmers. The benefits of exploiting the morphology of soft robots and optimizing it to simplify the control are further discussed in the upcoming modeling and control sections.

Fabricating and assessing these designs are also challenging processes due to the traditional manufacturing methods being unsuitable for these unconventional soft material. Additive manufacturing (AM) is one of the impactful technologies that helped enable this process [48,49]. One approach is to use AM to only fabricate the mold that would be used to pour the soft material in them. A more hybrid approach takes advantage of AM techniques, such as the fused filament fabrication (FFF) method, in addition to molding techniques to fabricate and assemble complex soft robotic systems. The third approach is the total additive manufacturing (TAM) approach. It exploits all the benefits of AM to fabricate soft robots, whether by 3D printing multiple soft parts and assemble them, or manufacturing the complete soft robot as a whole part. Such advance in 3D printing techniques for soft materials increased the ability to produce and test different designs of soft robots and optimize their morphological and material parameters.

### 3.2. Actuation

The actuation of soft robots poses several challenges due to the large number of degrees of freedom resulting from the large deformation of the soft materials that constitute them, making them underactuated systems that are harder to control. In addition, most conventional robotics actuators, such as DC motors, are bulky and rigid, which contradicts the main reason for developing soft robots with high compliance. Nonetheless, some soft robots use servo motors and gear pumps for fluidic actuation, while others use more unconventional actuators such as smart actuators, chemical reactions, and stiffness modulation [50].

One common actuation method is the use of tendon wires that are anchored at several points in the body of the soft material. These cables are driven by applying tension to them using electric motors such as servos, causing the connected soft material to deform, resulting in different motions or shape changes of the soft body. One example is the bioinspired octopus’s arm [51] made of silicone that is driven using cables. It can perform crawling motion and grasping similar to actual octopus tentacles. The use of traditional motors provides a large actuation force, especially in underwater applications where a powerful enough thrust is needed for locomotion. The shape deformation can be approximately determined through the displacement of the anchoring points of the cables.

Fluidic Elastomer Actuators (FEAs) is another type of soft actuators that rely mainly on fluid pressure [52]. The actuators are made from hyperelastic materials with embedded channels that expand due to the applied pressure. One of the early implementations is the Pneumatic Artificial Muscles (PAMs), most notably the McKibben artificial muscle actuator [53,54], which is made from a flexible elastomer tube constrained by a reinforced fiber to limit its extension but allow it to expand when pressurized, providing considerable force. Other types of fluidic elastomers use various means of pressurization, including pneumatic sources using compressed air [55,56], pressurized gas such as CO_2_ [57,58,59], or chemical pressure generation [60,61], as well as hydraulic sources [62,63,64]. The multigait crawling robot [55,57] has pneumatic actuators with a Pneu-Net (PN) architecture. The PNs are composed of a series of extensible chambers that inflate when pressurized and an inextensible layer that constrains the expansion of the chambers, causing the elastomer to bend. The geometrical parameters of the chambers and the constraining layer guide the deformation of the elastomer, affecting its bending and twisting motion. Underwater applications using fluidic elastomers include a biomimetic autonomous fish with a bidirectional pneumatic elastomer [59], an extended version of the former fish using a hydraulically pressurized elastomer instead [63], and an underwater crawling robot having bellow fluidic actuators as legs [64]. The completely soft Octobot [61] relies on totally soft microfluidic logic to control gas generation through chemical fuel decomposition, causing actuation. The use of fluidic actuators is advantageous for obtaining high material deformation and the ability to arrange actuators in an agonist-antagonist form, similar to muscle pairs. However, they are slow and have delayed response, and their pressurization units can be hard to embed inside soft robots.

Another actuation approach is the use of different types of smart materials. Smart materials are distinct in their response to external thermal or electric stimuli, causing deformation or stiffness change to the material. Electroactive polymers (EAPs) use electric stimuli to deform. Dielectric elastomer actuators (DEAs) are a type of EAPs that comprise two compliant electrodes that are compressed when high voltage is applied to them [65]. Compression force can be used to induce motion [66,67]. Another type of EAPs used for soft robots’ actuation is ionic polymer metal composite (IPMC). It is composed of Nafion polymer and electrodes. Applying voltage to the electrodes causes the polymer to deform due to the ionization process and the motion of ions between the two electrodes [68,69,70]. Shape memory alloys (SMAs) are smart materials that react to heat stimuli. When applying high temperature to the SMA, it deforms into a certain shape and is restored to its original shape after heat is removed. The heat is usually provided through electrical heating using high voltage. SMAs are used as actuators in soft robotics, as they can be embedded to drive a soft material such as polydimethylsiloxane (PDMS) [71,72].

The use of smart actuators is prominent in underwater robotics [73] due to the favorable operating conditions for smart materials in water. In addition, smart materials can be directly embedded within the elastically deformable body of the robots, making them a good option for biomimetic applications. For example, biomimicry of jellyfish was implemented using DEAs [67] and using SMAs in the case of Robojelly [74]. Manta ray biomimetic robots were actuated using IPMCs [68] as well as SMAs [75]. A biomimetic crawling starfish used actuated legs made from embedded SMA wires cast in PDMS [71]. Another group developed a soft robotic arm inspired by octopus tentacles using cables and SMA springs [72]. The SMA springs help mimic the muscular hydrostat of the octopus’s arm by providing transversal contraction. Smart actuators provide an advantage in terms of their compact size and weight, and high actuation biomimicry resembling real fish swimming modes. However, they require high-voltage sources and are hard to control. The various soft robotic platforms are shown in Table 1, classifying their biomimetic inspiration, actuation types, swimming modes, and level of compliance.

### 3.3. Modeling

**Figure 2:**
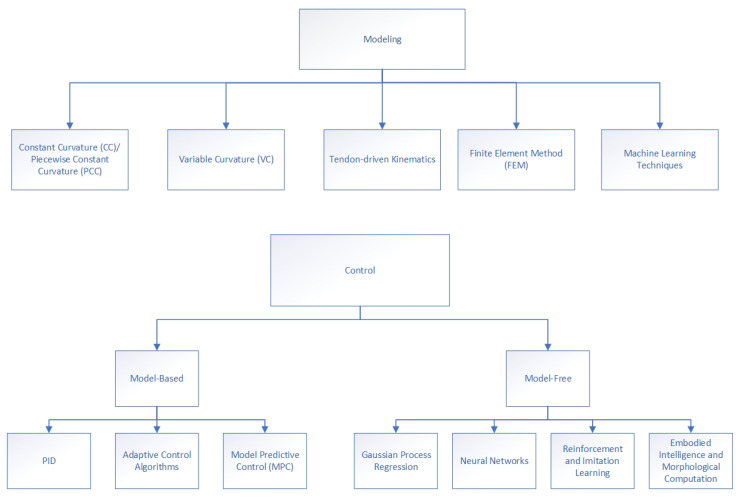
An overview of the different modeling and control techniques used in soft robotics.

The modeling and control phase is the most challenging part of building functional soft robotic systems capable of performing complex tasks and intelligently interacting with their own environment. All the well-established modeling techniques for rigid robots cannot be applied to soft robots due to their continuum property and their complex non-linear dynamics inherent from their elastic behavior. As conventional kinematic and dynamic modeling methods are inapplicable, new approaches for modeling and control of soft robots are being developed (Figure 2).

These modeling approaches can be separated into two main categories: model-based approaches and model-free data-driven methods. While the former relies on formulating either an exactly accurate or a simplified approximate model of a soft robot, the latter tries to implicitly learn the behavior of the robot using input and output data collected directly from the actual system.

The main goal of the modeling process is to map the soft robot’s actuation space to the configuration or task space. Since continuum soft robots are infinite-dimensional systems, formulating such models becomes highly difficult. Instead, the modeling methods rely on approximations and assumptions to reduce the system to a finite-dimensional one. The most commonly used simplification for kinematic modeling is the constant curvature (CC) method [84]. It assumes that the soft robot has constant strain along its whole length [85]. The piecewise constant curvature (PCC) method is an extended version of the CC that assumes the strain to be piecewise constant, with each segment of the soft structure having a constant strain [86]. A further extension is the variable curvature (VC) [87,88], which models each section as a CC. The PCC approximation is mainly valid for cable-driven soft manipulators. However, it cannot capture all the complex dynamics of soft structures, as it is a steady-state model. Nonetheless, it proves to be an accurate model for control for a lot of tendon-driven manipulators [89,90,91,92]. One group also used a PCC kinematic model of a multi-segment pneumatic actuator to develop a dynamic motion controller [93].

Numerical techniques have also been used for modeling soft robots. The Cosserat rod theory is one of these numerical methods [94,95], accurately representing the tension, shearing, bending, and torsion of rods. A different numerical approach for modeling soft robots is the finite element method (FEM), which yields more accurate results but at the cost of high computational requirements [96]. The method relies on discretizing the structure into a large number of nodes, called mesh, and iteratively solving the differential equations governing the behavior of these nodes, until the model converges. However, the use of FEM for real-time control is difficult, so control approaches based on real-time FEM have been proposed [97,98,99]. An approach combining kinematic modeling using PCC and Denavit–Hartenberg (DH) parameters with FEM analysis was also used to model a soft pneumatic actuator (SPA) [100]. The problem with the FEM is the high dimensional space of the obtained model. A common solution is to reduce the domain of the model in order to achieve high computational efficiency, without sacrificing accuracy [101,102]. The reduced-order model can help with the development of low-order controllers and observers based on a linearized model of the system [103,104]. One group developed a dynamic simulation tool for articulated soft robots based on numerical simulation methods for slender structures [105]. Another method uses genetic algorithms for dynamic parameters estimation of an octopus-inspired robot [106].

Controllers developed from the static kinematic models obtained using the described methods are considered static controllers that discard the underlying dynamics of the system. Developing high-order dynamic models for soft robots is difficult and computationally expensive for controllers. A first-order dynamic modeling approach is proposed to reduce the computational space without affecting the controller’s performance [107].

The model-free methods for modeling soft robots mainly use data-driven machine learning and deep learning techniques to find a mapping between the inputs and outputs of the soft system [108]. Input actuation signals and robot states can be obtained by sensors, either embedded or external visual tracking sensors. The data can then be used with different supervised learning, unsupervised learning, and reinforcement learning techniques to develop models and controllers for soft robots. Examples of commonly used techniques include feedforward neural network (FNN) [109,110,111,112], recurrent neural network (RNN) [113], convolutional neural network (CNN) [114], and echo state network (ESN) [109], based on the reservoir computing framework. FNNs are widely used for modeling soft systems. One approach is the use of an FNN to model the work envelope of an SPA with a variable inclination angle [115]. Another group used FEM-generated training data to learn the kinematic model of a 3D motion SPA using an FNN [111]. An RNN used sensory data from cPDMS resistive sensors and a load cell to predict the deformation and force models of a soft pneumatic finger [113]. A reservoir computing approach with the ESN architecture was used to model the 2D motion of a bioinspired turtle actuated through soft pneumatic flippers [109].

Learning-based techniques also proved to be successful in learning the dynamic models of soft robots. One approach involved using a nonlinear autoregressive exogenous (NARX) model to develop a dynamic model for a soft manipulator, which was used to implement a task space controller [116]. A deep neural network (DNN) model learned the non-linear dynamics for a single degree of freedom inflatable pneumatic robot. The model was used to implement a model predictive control (MPC) algorithm for pressure control [117].

### 3.4. Control

Models of soft robots developed using the discussed approaches are used to develop kinematic and dynamic control for these complex systems. Model-based controllers rely on models obtained from analytical kinematic methods such as PCC, whereas model-free controllers use data-driven techniques [91]. Different control algorithms are used, depending on the level at which the controller operates. Low-level controllers drive the actuators, whereas mid-level ones are responsible for the kinematic and dynamic control, and high-level control involves advanced trajectory and path planning for tasks such as obstacle avoidance.

The main task of soft robots’ controllers is to manage the whole-body deformation of the robot; it may also include controlling the exact position and orientation of an end effector in the case of soft manipulators. One approach uses open-loop control for a soft manipulator [118]. The implemented open-loop dynamic controller uses a data-driven model with only mechanical feedback. The swimming eel-like robot [119] was modeled using the geometrically exact beam theory with a torque control algorithm. A different approach uses an energy-shaping approach to develop the control law for a soft continuum manipulator [120]. MPC is another technique that was employed for the control of large-scale soft robots [121]. The MPC algorithm relies on a PCC model alongside a kinematic representation for efficient state prediction. A model reference predictive adaptive control (MRPAC) was also implemented on the same model and showed robustness to model uncertainties. Closed-loop control methods have also been demonstrated for the position control of soft robots [122,123].

One of the most promising approaches for the model-free control in the robotics field, in general, is reinforcement learning (RL), which has been proven successful for soft robots [124]. RL can be described as an iterative learning process where the agent takes an action, its new state is observed, and a response in the form of a reward function is given to it based on the resulting interaction with its environment. When the agent learns a policy to map appropriate state–action pairs, then the learning is successful. RL can be implemented regardless of whether a model of the system is known. The use of deep reinforcement learning (DRL) and imitation learning algorithms in the development of soft robots has been shown in several experimental examples. DRL methods are now integrated with soft robots in various applications such as biomedical and edible robotics. Some research focused on the deep Q-network (DQN) algorithm, which was used in a soft robotic fish used for underwater exploration [40]. Other common algorithms are deep deterministic policy gradient (DDPG), normalized advantage function (NAF), and advantage actor-critic (A2C). However, problems arise from the differences between the simulation environments where the robots are trained and the real-world environment. The use of generative adversarial network (GAN) is a suggested solution to help perform domain adaptation and narrow the gap between the simulation and real-world environments. Imitation learning is also beneficial when it is difficult to formulate a reward function to train a DRL model. The imitation learning algorithms use demonstrations constructed by an expert agent that are transferred to the soft robot. The most common imitation learning approaches are behavior cloning, inverse reinforcement learning, and generative adversarial imitation learning. The future scope is the combination of both deep reinforcement learning and imitation learning algorithms to benefit from both approaches and train better agents.One example is a soft artificial-muscle-driven robot mimicking cuttlefish actuated by a dielectric elastomer (DE) membrane [66]. The robot uses reinforcement learning to provide the actuation. To simplify the problem, only two actions are considered, with only two voltage amplitudes: 0 and 6.8 kV. The robot is trained through trial-and-error interaction with its environment in order to find an optimal policy to maximize its reward function, which is the displacement at each time step. The displacement is monitored using a camera and then fed to the reinforcement learning algorithm.

Applying reinforcement learning with soft robots is a costly operation due to their non-linear hyperelastic properties. To overcome this issue, the reinforcement learning approach used to control the Honeycomb Pneu-Nets Soft Robot ignores the specific properties of the materials and the structural characteristics of the robot [125], concentrating only on the geometric model, which simplifies the modeling task. Another challenge to implementing RL is the lack of accurate simulators for soft robots. To overcome this problem, researchers performed the training process on both the simulation as well as the physical hardware to obtain a more realistic control policy that works well with the actual robot. The reinforcement learning framework consists of two parts: formulating the set of representations for the robot’s states and actions, and the training process to search for accurate results in the problem space. The physical hardware uses air pumps and valves to achieve the actuation needed and uses the OptiTrack motion capture system for visual sensing to determine the actual state of the robot. Two methods of control were used to execute the learned RL policy: open-loop and closed-loop. The closed-loop method obtains the actual robot state from the sensor as the input to the trained control policy function, while in the open-loop method, the robot’s state is obtained from the simulator, leading to some errors.

Despite providing a good solution in many cases, RL may be impractical to implement when the reward function canonot be clearly defined. Imitation learning solves this issue by using demonstrations performed by an expert agent. One group used imitation learning to perform motion control and trajectory planning for soft continuum robots [126]. They proposed the learning from demonstration (LfD) approach and implemented it on the Bionic Handling Assistant (BHA) robot, which resembles an elephant trunk. One of the simplest methods to implement LfD is kinesthetic teaching, which is achieved by directly recording demonstrations on the target robot to collect the position and orientation data of the end effector. The problem of using kinesthetic teaching with soft robots arises from the dynamic complexity of non-linear elasticity associated with these soft materials. They proposed an active compliant control to record the demonstrations during kinesthetic teaching, then encoded the recorded data with a task-parametrized Gaussian mixture model (TP-GMM).

A similar approach was used with the soft cylindrical robot arm STIFF-FLOP [127]. However, the researchers worked on transferring the movement patterns of an octopus arm to the STIFF-FLOP. The octopus arm movements were obtained from a database of the cartesian position of several octopuses’ arms with an average of 100 points along the arm. They exploited several methods for the representation of the octopus reaching motion to help transfer the arm movements to the soft robot. They considered spatiotemporal representation and dynamical movement primitives to allow for a more robust movement transfer. They then used Gaussian mixture regression (GMR) for encoding, and applied a self-refinement algorithm with a weighted reward function according to different tasks.

Following a similar thought process to imitation learning, a trending new paradigm called morphological computation aims to learn from living creatures, but on the level of morphology instead of the locomotion level. Morphological computation is a process related to embodied intelligence, where some of the computation needed for actuation and perception is conducted automatically by the body of the living creature instead of sending sensory information to the brain and waiting for a control signal [128]. Morphological computation can be exploited in the context of soft robots to offload some of the control to the body. This process is possible owing to the adaptable compliance exhibited by soft materials, which act as a reservoir computer that can process inputs from its environment and take appropriate actions.

A morphological computation framework with a mathematical realization was proposed using a reservoir of recurrent non-linear mass-spring systems, which is a model for actual physical soft bodies [129]. The model demonstrated the ability to learn the end-effector trajectory of a robotic arm. The group also showed that adding feedback to the morphological computation system allows it to perform autonomous periodic patterns such as the ones responsible for locomotion [130].

## 4. Prospective Directions

Soft robotics is still a new field with many challenges and obstacles, but shows promising potential. Current research is aimed toward the establishment of a unified foundation and framework for the process of building complete and functional soft robots, the same as the basis available for conventional rigid robots.

One promising approach is the attempt to automate and optimize the various aspects of soft robots’ development, including the design, manufacturing, choice of actuation, and sensing components. The exploration of new soft materials with variable controlled stiffness, suitable for different environments, and the exploitation of their morphology in actuation, sensing, and control may provide several solutions to the current challenges. The use of rapid design and manufacturing processes and of the technological advances in additive manufacturing is needed to allow the development of complex soft robotic systems. Another approach is to develop completely soft robots, void of any type of rigid elements. All actuators, sensors, and processing units will need to be implemented using completely soft materials. In addition, the development of general modeling and control techniques that can be used for all types of soft robots would strongly boost advances in the field.

Embodied intelligence provides a possible solution for building soft robots that are close to their biological inspirations. Similar to the connection between the brains and bodies of living creatures, where the morphology of the body takes part in the interaction with the environment, soft robotic systems may be built to exploit their compliance in the computation and processing of data they receive while navigating and adapting to their surroundings. Another potential direction is the investigation of using organic living tissues to help build soft robotic systems. Since soft robots try to mimic actual living creatures, the use of organic parts to fabricate soft robots would be beneficial. These living tissues may be embedded in soft robots to exploit their biological actuation and sensory features. In addition, it is important to develop soft sensors that can easily be embedded within the structures of soft robots, without significantly affecting their compliance. This would enable proprioceptive sensing of the robots’ deformation, allowing better control and more interaction with the environment. Finally, taking more inspiration from biological systems while developing soft robots, by integrating all the robotic components from brain to morphology, to actuation, and sensing, into homogeneous entities may help with the development of more biological-like systems.

## Figures and Tables

**Table 1:** Classification of various underwater soft robotic systems.

Reference	Robot	Biomimicry	Actuation	Swimming	Compliance
[37]	Multi-Joint Fish	Carangiform Fish	Electric Actuators (Servomotors)	BCF Undulation	Medium
[69,70]	Biomimetic Fish	Fish	IPMC	BCF/MPF Oscillation	Medium
[40,59,63]	SoFi	Fish	FEA (Pneumatic/Hydraulic)	BCF Undulation	High
[41]	Stingray Robot	Stingray	Electric Actuators (Servomotors)	MPF Undulation	Medium
[51]	Octopus Arm	Octopus	Motor-driven Cables	Crawling	High
[72]	Octopus Arm	Octopus	Motor-Driven Cables/SMA Springs	-	High
[76]	Octopus Robot	Octopus	Motor-Driven Cables/SMA	Crawling	Medium
[66]	Cuttlefish Robot	Cuttlefish	DEA	Jet Propulsion	Medium
[74]	Robojelly	Jellyfish	SMA	Propulsion	High
[61]	Octobot	Octopus	FEA (Chemical Reaction)	-	High
[64]	Morphing Underwater Walking Robot	-	FEA (Hydraulic)	Walking/Crawling	Medium
[67]	Jellyfish-Inspired Soft Robot	Jellyfish	DEA	Propulsion	High
[69]	Robotic Manta Ray	Manta Ray	IPMC	MPF Undulation	Medium
[75]	Micro Biomimetic Manta Ray	Manta Ray	SMA	MPF Undulation	Medium
[71]	Starfish Robot	Starfish	SMA Wires	Propulsion	High
[77]	Starfish-Like Soft Robot	Starfish	SMA	Crawling	High
[78]	RoboScallop	Scallop	FEA	Jet Propulsion	Medium
[79]	Eel-like Robot	Leptocephalus (Eel Larva)	Fluid Electrode DEA (FEDEA)	BCF Undulation	High
[80]	Morphing Limb Amphibious Turtle Robot	Turtle/Tortoise	Variable Stiffness Material-pneumatic Actuators	Drag-induced Swimming/Walking	Medium
[81]	FinRay Robotic Jellyfish	Jellyfish	FinRay Actuators driven with Servomotors	Propulsion	Medium
[82]	PATRICK: Brittle Star-Inspired Soft Robot	Brittle Star	SMA Wires	Crawling	High
[83]	Soft Underwater Starfish	Starfish	Servo-driven Tendon Wires	Propulsion	High
